# Beyond static frailty: longitudinal trajectories, nonlinear associations, and muscle strength mediation in osteoporosis risk

**DOI:** 10.7189/jogh.16.04320

**Published:** 2026-10-19

**Authors:** Junyu Zhou, Jui-Hua Wang, Zhen Huang, Liping Cao

**Affiliations:** 1 Department of Rheumatology and Immunology, Xiangya Hospital, Central South University, Changsha, China; 2 Provincial Clinical Research Center for Rheumatic and Immunologic Diseases, Xiangya Hospital, Changsha, China; 3 Department of Dermatology, Hunan Engineering Research Center of Skin Health and Disease, Hunan Key Laboratory of Skin Cancer and Psoriasis, Xiangya Hospital, Central South University, Changsha, China; 4 National Clinical Research Center of Geriatric Disorders, Xiangya Hospital, Central South University, Changsha, China; 5 Hunan Yueya Dental Clinic, Changsha, China; 6 Changsha Chang Hao Hospital, Changsha, China; 7 Department of Emergency, Xiangya Hospital, Central South University, Changsha, China

**Keywords:** Frailty Index, osteoporosis, trajectory analysis, group-based trajectory modelling, grip strength, longitudinal cohort

## Abstract

**Background:**

Frailty, a multidimensional indicator of declining physiological reserve, has been consistently linked to adverse health outcomes. However, whether dynamic trajectories of the Frailty Index (FI) predict new-onset osteoporosis remains unclear.

**Methods:**

For this longitudinal cohort study, we retrieved data on adults aged ≥50 years from the Health and Retirement Study, the English Longitudinal Study of Ageing, and the Survey of Health, Ageing and Retirement in Europe. We assessed frailty using the FI and identified longitudinal frailty trajectories, examined associations of baseline FI and frailty trajectories with incident osteoporosis, and explored nonlinear relationships and the potential mediating role of grip strength.

**Results:**

Over median follow-up periods of 4, 6, and 8 years, 244, 2,693, and 1,986 participants in the ELSA, the SHARE, and the HRS cohorts developed osteoporosis, respectively. Higher baseline FI was independently associated with greater osteoporosis risk across all cohorts, with adjusted hazard ratios of 1.43 (95% confidence interval (CI) = 1.36–1.50, *P* < 0.001) in the HRS, 1.36 (95% CI = 1.19–1.57, *P* < 0.001) in the ELSA, and 1.39 (95% CI = 1.33–1.45, *P* < 0.001) in the SHARE cohort. We identified four FI trajectories. Compared with the low frailty group, participants in higher frailty trajectory groups generally exhibited increased risks of osteoporosis across cohorts. Nonlinear analyses pointed to threshold effects in the HRS and the SHARE, with steeper risk increases at lower FI levels. Grip strength partially mediated this association.

**Conclusions:**

In our sample, baseline FI and its longitudinal trajectories were significantly associated with osteoporosis. Our findings suggest that the early identification and monitoring of frailty, as well as muscle strength improvement, may help reduce osteoporosis risk.

Osteoporosis is a systemic skeletal disorder that manifests in reduced bone mineral density and deterioration of bone microarchitecture, leading to a substantially increased risk of bone fractures [1]. Although global age-standardised fracture rates have declined slightly, the absolute number of fractures is projected to increase markedly, with this rise in prevalence largely related to the phenomenon of population ageing [2]. Hip and vertebral fractures, which are the most common and severe manifestations of osteoporosis, often result in long-term disability, increased mortality, and substantial reductions in health-related quality of life [3]. The associated medical and socioeconomic burden is considerable, with direct healthcare costs reaching billions of dollars annually and an increasing demand for long-term care. Therefore, early identification of individuals at high risk of osteoporosis is a major public health priority [4].

Frailty, meanwhile, is a clinical syndrome characterised by diminished physiological reserves and increased vulnerability across multiple organ systems. It is strongly associated with adverse outcomes, including falls, progressive disability, and premature mortality [5]. A growing base of longitudinal evidence suggests that frailty shares common biological pathways with osteoporosis and fracture risk [6]. Proposed mechanisms include sarcopenia-related muscle loss, chronic low-grade inflammation, and endocrine dysregulation involving sex and parathyroid hormones [7]. The Frailty Index (FI), which is based on the accumulation of health deficits, is an exposure metric that provides a quantitative and continuous measure of multisystem vulnerability. It is particularly valuable due to its reproducibility and ability to capture graded risk, while its predictive performance for osteoporotic fractures has been shown to be comparable to or even exceed established risk prediction tools such as Fracture Risk Assessment Tool [8,9].

Despite the growing evidence linking frailty with adverse skeletal outcomes and the increasing use of the Frailty Index (FI) in ageing research, several important gaps remain. Most existing studies have relied on a single FI measurement, which limits the ability to capture dynamic changes in frailty over time [9]. Few studies have applied group-based trajectory modelling (GBTM) to characterise the heterogeneity in the progression of frailty and its differential effects on bone health [10]. Evidence regarding nonlinear dose–response relationships or threshold effects between FI and new-onset osteoporosis also remains limited. Furthermore, the underlying mechanisms, particularly the potential mediating role of muscle strength, have not been adequately investigated in prospective settings [11,12]. Lastly, most studies remain based on single-country or single-cohort data, restricting the generalisability of their results across regions and populations [13–15].

To address these limitations, we leveraged data from longitudinal cohorts of ageing populations from several countries to examine the prospective associations between the FI, its longitudinal trajectories, and the risk of new-onset osteoporosis. Distinct frailty trajectories are identified using group-based trajectory modelling. Multiple complementary analytical approaches, including Cox proportional hazards models, competing risk models, and propensity score methods, are employed to ensure robustness. Restricted cubic spline models and segmented regression are used to explore nonlinear relationships and potential threshold effects. External validation across multiple cohorts further enhances the generalisability and translational relevance of the findings.

## METHODS

We retrieved data on community-dwelling older adults captured in three longitudinal cohorts: the Health and Retirement Study (HRS), the English Longitudinal Study of Ageing (ELSA), and the Survey of Health, Ageing and Retirement in Europe (SHARE). The three cohorts were nationally representative of the USA, the UK, and multiple European countries, respectively, which differ in demographic composition, healthcare systems, socioeconomic structures, and approaches to osteoporosis detection and management. Within these cohorts, data on participants’ sociodemographic characteristics, health status, functional status, and health-related behaviours were gathered through biennial standardised interviews and physical examinations.

We included participants who were aged 50 years or older at baseline, had sufficient information available for FI construction (≤20% missing FI deficits), were free of osteoporosis at baseline, and had follow-up information available for the ascertainment of incident osteoporosis.

### Assessment of the FI and construction of frailty trajectories

We constructed the FI using a deficit accumulation approach based on the framework proposed by Rockwood and Mitnitski [16,17]. This approach has been extensively validated as a measure of biological ageing and vulnerability to adverse health outcomes and is one of the most widely used operationalisations of frailty in epidemiological research. Consistent with the original methodology proposed by Mitnitski and colleagues and subsequently refined by Rockwood and colleagues [16,17], we conceptualised frailty as the cumulative burden of age-related health deficits selected according to biological relevance, association with adverse health outcomes, increasing prevalence with age, and avoidance of early saturation in the general population. The selected variables were considered representative of these criteria and spanned multiple domains, including chronic diseases, functional limitations, activities of daily living, self-rated health, cognitive function, and psychological status.

We selected the deficit accumulation approach, rather than phenotype-based frailty measures, because it is particularly well suited for harmonised analyses across multinational longitudinal cohorts [16–18]. The FI incorporates information from multiple health domains, can accommodate differences in available variables across cohorts through standardised harmonisation procedures, and provides a continuous measure that is advantageous for trajectory modelling and dose–response analyses. By contrast, alternative frailty operationalisations, such as the Fried frailty phenotype, rely on specific physical performance assessments that were not consistently available across all cohorts and study waves.

We coded all deficit variables on a scale from 0 to 1, where 0 indicated absence and 1 indicated full presence of a deficit. Ordinal variables were proportionally coded according to severity, whereby a five-level self-rated health variable was coded as 0 (excellent), 0.25 (very good), 0.50 (good), 0.75 (fair), and 1.00 (poor). We applied similar proportional coding procedures to other ordinal deficit variables, with intermediate categories assigned values between 0 and 1 according to their relative severity.

We calculated the FI as the ratio of the number of deficits present to the total number of deficits considered. For participants meeting the inclusion criteria, we determined FI scores using all available deficit information according to the standard deficit accumulation approach. No additional imputation procedures were applied. Participants with excessive missing deficit data (>20%) were excluded prior to FI construction to ensure that the estimates would be reliable. We treated FI as a continuous variable in the primary analyses and further categorised it into cohort-specific quartiles in the descriptive and survival analyses; in both cases, higher FI values indicated greater frailty. We multiplied FI values by 100 and expressed them as percentages, which we then used in all regression analyses, including Cox, Fine–Gray, and discrete-time logistic regression models. This means that all estimates correspond to a one-percentage-point increase in FI.

To ensure comparability, we harmonised the data across cohorts prior to FI construction using a previously validated deficit accumulation framework that has been widely applied in HRS, ELSA, and SHARE studies [19–22]. In this process, conceptually equivalent variables were identified and recoded using standardised definitions across cohorts whenever possible (Table S1 in the **Online Supplementary Document**).

We constructed frailty trajectories to capture heterogeneity in longitudinal changes using the FI measurements from the first three consecutive waves of the final analytical cohorts described above. For this purpose, we created a GLBM model with time defined as an independent variable number of years since baseline, with approximately two-year intervals between waves, and with FI as the dependent variable. We fitted models with varying numbers of trajectory groups and selected the optimal one based on both statistical fit and clinical interpretability. Each participant was assigned to a trajectory group according to the maximum posterior probability. This approach lent itself to a balance between an accurate characterisation of early longitudinal frailty patterns and an adequate subsequent follow-up for the assessment of incident osteoporosis, and ensured that frailty trajectories were established before outcome occurrence, which strengthened the temporal ordering and reduced the potential for reverse causation.

Frailty trajectories were derived from the final analytical cohorts; no additional participant selection was performed specifically for GBTM. The trajectory analyses included 14,917 participants in the HRS cohort, 7,345 participants in the ELSA cohort, and 35,678 participants in the SHARE cohort. The identified trajectory groups were subsequently treated as categorical exposures in the analysis of new-onset osteoporosis risk.

### Assessment of new-onset osteoporosis

The primary outcome was new-onset osteoporosis. Osteoporosis status across cohorts was determined based on self-reported physician diagnosis, whereby participants were asked during follow-up interviews whether a physician had ever informed them that they had osteoporosis. Although the exact wording differed slightly across studies, all cohorts used comparable self-reported physician-diagnosed osteoporosis measures. Incident osteoporosis was defined as the first report of physician-diagnosed osteoporosis during follow-up among participants who were free of osteoporosis at baseline. Follow-up assessments were conducted approximately every two years in the HRS, ELSA, and SHARE cohorts. Follow-up time was calculated from baseline to the earliest occurrence of osteoporosis, loss to follow-up, or end of follow-up. Participants who did not develop osteoporosis were censored at the end of follow-up. Outcome definitions were harmonised across cohorts to maximise comparability.

We included the following set of variables as covariates, harmonising them where possible to ensure comparability across the three cohorts: age, sex, race, education, wealth, body mass index (BMI), hypertension, diabetes, smoking status, drinking status, physical activity, cancer, and cardiovascular disease. We harmonised smoking and drinking status as binary variables (never *vs*. ever smokers and never *vs*. ever drinkers, respectively) to ensure comparability across cohorts. We handled missing covariate data using multiple imputation by chained equations, generating five imputed data sets using and combining estimates according to Rubin’s rules. Participants with missing data for covariates included in a given multivariable model were excluded from that specific analysis using a complete-case approach.

### Statistical analysis

We assessed the continuous variables for the normality of distribution using the Shapiro–Wilk test and visual inspection of histograms with kernel density curves (Figure S2 in the **Online Supplementary Document**). We summarised variables with approximately normal distributions as means with standard deviations and compared them using Student’s *t*-tests. For variables with non-normal distributions, we presented them as medians with interquartile ranges and compared them using the Mann-Whitney U test. We otherwise presented categorical variables as counts and percentages and compared them using the chi-squared tests.

We then used GBTM based on finite mixture models to identify distinct FI trajectories. This approach classifies individuals into latent subgroups with similar developmental patterns using maximum likelihood estimation. Analyses were conducted separately for each cohort using FI data from the first three waves. We evaluated models with one to four trajectory groups and selected the optimal one based on the Bayesian information criterion, Akaike information criterion, log-likelihood values, and mean posterior probabilities, as well as a requirement that each trajectory group included >5% of the total analytical sample. Trajectory solutions were also evaluated based on whether the identified groups represented clinically meaningful patterns of frailty progression and whether comparable trajectory patterns were observed across cohorts.

We examined associations between FI, FI trajectories, and new-onset osteoporosis using multivariable Cox proportional hazards models, with results presented as hazard ratios (HRs) and 95% confidence intervals (CIs). We defined the time-to-event from the end of the trajectory assessment period to the occurrence of osteoporosis, censoring, or study end. To evaluate the robustness of the observed associations, we set up four models: an unadjusted model (model 1), a model adjusted for age, sex, race, education, marital status, wealth, and BMI (model 2), a model further adjusted for hypertension, diabetes, smoking status, and drinking status (model 3), and a model yet further adjusted for cardiovascular disease and cancer (model 4).

Given that death may preclude the occurrence of osteoporosis, we performed competing risk analyses using the Fine–Gray subdistribution hazard model, with death treated as a competing event. The results of this modelling were reported as subdistribution hazard ratios (sHRs) and 95% CIs. To further assess robustness, we applied discrete-time logistic regression models using follow-up waves as the time unit, restructuring data into a person-period format, with each participant contributing observations until osteoporosis onset or censoring.

We conducted restricted cubic spline analyses to evaluate potential nonlinear associations between FI and new-onset osteoporosis. When evidence of nonlinearity was observed, we evaluated threshold effects using two-piecewise linear regression models, where we determined the optimal inflection point through a recursive search procedure that identified the value associated with the maximum model likelihood. Model fit between the standard linear model and the two-piecewise linear model was compared using a likelihood ratio test. To explore potential mechanisms underlying the association between frailty and new-onset osteoporosis, we performed causal mediation analyses in the HRS and SHARE cohorts using the ‘mediation’ package in *R*. Grip strength was selected *a priori* as the mediator based on its established role as an indicator of muscle function and its potential contribution to the relationship between frailty and skeletal health. The FI was specified as the exposure, grip strength as the mediator, and new-onset osteoporosis as the outcome. We estimated the average causal mediation effect, average direct effect, total effect, and proportion mediated after adjustment for the same covariates included in the fully adjusted model. Statistical inference was based on nonparametric bootstrap resampling with 5,000 simulations, for which we calculated 95% CIs. These analyses assume no substantial unmeasured confounding of the exposure–mediator, exposure–outcome, or mediator–outcome relationships after adjustment for measured covariates. We performed subgroup analyses to assess the consistency of the obtained results across populations. We conducted all analyses using SPSS, version (IBM, Armonk, New York, USA) and *R*, version 4.3.0 (R Core Team, Vienna, Austria). A two-sided *P*-value <0.05 was considered statistically significant.

## RESULTS

We identified 88,202 participants across the three cohorts. After excluding ineligible individuals (Figure S1 in the **Online Supplementary Document**), we retained data on 57,940 participants in our analytical sample.

The median baseline age for the 14,197 participants in the HRS cohort, of whom 53.76% were women, was 64 years (Tables S2 in the **Online Supplementary Document**). During a median follow-up of 8 years, 1,986 participants with a baseline median age of 67.00 years developed osteoporosis. Compared with those who remained osteoporosis-free, participants who developed osteoporosis had an older baseline age (*P* < 0.001), a higher proportion of women (*P* < 0.001), a higher proportion of smokers (*P* < 0.001), lower muscle strength (*P* < 0.001), and higher FI values (*P* < 0.001).

The median baseline age of the 7,345 participants of the ELSA cohort, of whom 52.70% were women, was 66 years (Table S3 in the **Online Supplementary Document**). Over a median follow-up of 4 years, 244 participants with a median baseline age of 69.50 years developed osteoporosis. Compared with those without osteoporosis, affected individuals were older (*P* < 0.001), had a higher proportion of females (*P* < 0.001), had lower household wealth (*P* = 0.002), and had higher FI values (*P* < 0.001).

The median baseline age of the 35,678 participants in the SHARE cohort, of whom 51.63% were women, was 64 years (Table S4 in the **Online Supplementary Document**). During a median follow-up of 6 years, 2,693 participants with a median baseline age of 68 years developed osteoporosis. Compared with those who did not develop osteoporosis, affected individuals were older (*P* < 0.001), predominantly female (*P* < 0.001), had lower household wealth (*P* < 0.001), lower BMI (*P* < 0.001), lower muscle strength (*P* < 0.001), and higher FI values (*P* < 0.001).

Compared with included participants, excluded participants were generally older, had higher frailty levels, lower physical activity, and a greater burden of chronic conditions across cohorts, which is suggestive of selective attrition during follow-up (**Online Supplementary Document**).

### Association between the FI, its trajectories, and risk of new-onset osteoporosis

In the unadjusted models, baseline FI was positively associated with osteoporosis risk across all cohorts. After full adjustment for confounders, baseline FI modelled on the conventional 0–1 scale remained significantly associated with new-onset osteoporosis, with adjusted HRs of 1.43 in the HRS (95% CI = 1.36–1.50, *P* < 0.001), 1.36 in the ELSA (95% CI = 1.19–1.57, *P* < 0.001), and 1.39 in the SHARE cohort (95% CI = 1.33–1.45, *P* < 0.001). Overall, each one-percentage-point increase in FI was consistently associated with a higher risk of new-onset osteoporosis across all cohorts (Table S6 in the **Online Supplementary Document**).

We identified four distinct FI trajectories using GBTM: low, moderate, high, and very high frailty (**Figure 1**; Table S7 in the **Online Supplementary Document**). Compared with the low frailty group, participants in higher frailty trajectory groups had progressively increased risks of new-onset osteoporosis after full adjustment (). Trend analyses confirmed a clear dose–response relationship. Kaplan-Meier analyses showed significant differences in osteoporosis-free survival across trajectory groups in all cohorts, with log-rank *P*-values <0.001 across the three cohorts. Survival probabilities declined more rapidly in groups with higher frailty trajectories (Figure S3 in the **Online Supplementary Document**).

**Figure 1:**
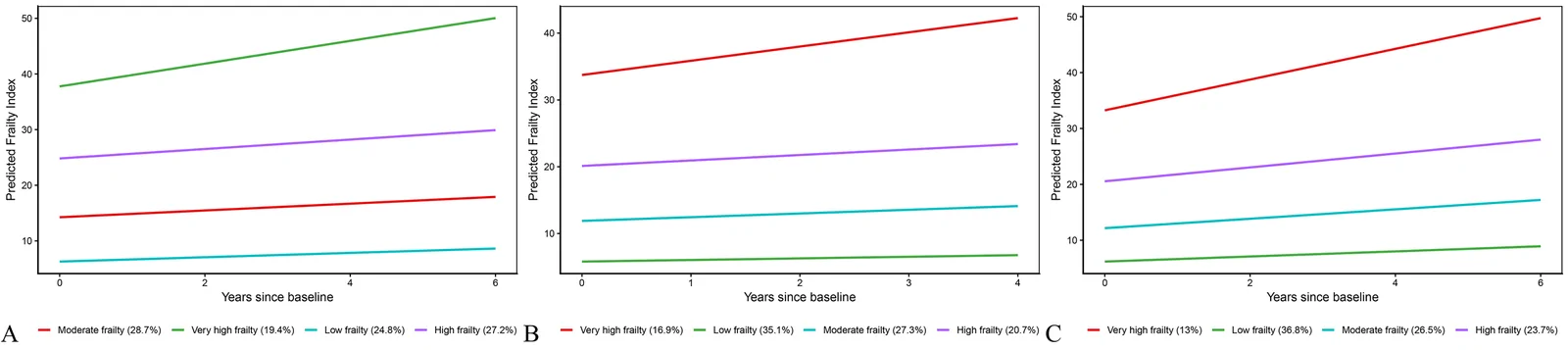
Trajectories of the frailty index identified using trajectory analysis in the study population. **Panel A.** HRS cohort. **Panel B.** ELSA cohort. **Panel C.** SHARE cohort.

### Competing risk analysis considering mortality

After adjustments in the Fine–Gray competing risk models (**Table 1**), each one-percentage-point increase in FI remained independently associated with a higher risk of new-onset osteoporosis, with adjusted sHRs of 1.41 in the HRS (95% CI = 1.35–1.48, *P* < 0.001), 1.36 in the ELSA (95% CI = 1.19–1.55, *P* < 0.001), and 1.31 in the SHARE (95% CI = 1.25–1.36, *P* < 0.001). When stratified by FI trajectories, participants in the moderate, high, and very high frailty groups exhibited significantly higher risks compared with the low frailty group. Trend tests were significant across all cohorts, with all *P*-values <0.001. The magnitude and direction of these associations were consistent with those observed in Cox models, indicating that competing mortality did not materially influence the findings. Notably, an irregular pattern was observed in the ELSA cohort, where the moderate frailty trajectory group exhibited a higher sHR than the high frailty trajectory group. Given the relatively small number of osteoporosis events in ELSA (n = 244), particularly after stratification into multiple trajectory categories, this finding may reflect statistical instability and should, therefore, be interpreted cautiously. The expected dose–response relationship was observed in the primary Cox analyses and in the larger HRS and SHARE cohorts.

**Table 1:** Fine–Gray regression analysis examining whether the associations between the FI, its trajectories, and the risk of new-onset osteoporosis are influenced by mortality*

	Model 1		Model 2		Model 3		Model 4	
	**sHR (95% CI)**	***P*-value**	**sHR (95% CI)**	***P*-value**	**sHR (95% CI)**	***P*-value**	**sHR (95% CI)**	***P*-value**
**HRS**								
Frailty	1.32 (1.28–1.37)	<0.001	1.34 (1.29–1.39)	<0.001	1.39 (1.33–1.45)	<0.001	1.41 (1.35–1.48)	<0.001
Frailty group								
*Low frailty*	ref		ref		ref		ref	
*Moderate frailty*	1.39 (1.21–1.61)	<0.001	1.52 (1.32–1.76)	<0.001	1.62 (1.39–1.88)	<0.001	1.63 (1.41–1.89)	<0.001
*High frailty*	1.92 (1.67–2.20)	<0.001	2.18 (1.89–2.53)	<0.001	2.43 (2.09–2.82)	<0.001	2.48 (2.13–2.90)	<0.001
*Very high frailty*	2.99 (2.61–3.43)	<0.001	3.38 (2.91–3.92)	<0.001	3.85 (3.29–4.50)	<0.001	4.02 (3.41–4.73)	<0.001
*P*-*value or trend*	1.59 (1.51–1.67)	<0.001	1.64 (1.55–1.74)	<0.001	1.72 (1.62–1.82)	<0.001	1.76 (1.65–1.87)	<0.001
**ELSA**								
Frailty	1.46 (1.34–1.59)	<0.001	1.36 (1.23–1.50)	<0.001	1.38 (1.24–1.54)	<0.001	1.36 (1.19–1.55)	<0.001
Frailty group								
*Low frailty*	ref		ref		ref		ref	
*Moderate frailty*	5.06 (3.47–7.36)	<0.001	4.12 (2.76–6.16)	<0.001	4.69 (3.08–7.15)	<0.001	4.79 (3.04–7.55)	<0.001
*High frailty*	1.73 (1.14–2.63)	0.010	1.61 (1.06–2.46)	0.027	1.76 (1.15–2.69)	0.009	1.79 (1.17–2.75)	0.007
*Very high frailty*	2.86 (1.92–4.27)	<0.001	2.37 (1.57–3.59)	<0.001	2.67 (1.75–4.06)	<0.001	2.75 (1.79–4.20)	<0.001
*P*-*value or trend*	1.92 (1.68–2.18)	<0.001	1.76 (1.53–2.03)	<0.001	1.83 (1.57–2.14)	<0.001	1.85 (1.56–2.19)	<0.001
**SHARE**								
Frailty	1.40 (1.36–1.43)	<0.001	1.29 (1.25–1.33)	<.001***	1.30 (1.26–1.35)	<0.001	1.31 (1.25–1.36)	<0.001
Frailty group								
*Low frailty*	ref		ref		ref		ref	
*Moderate frailty*	1.91 (1.69–2.15)	<0.001	1.86 (1.65–2.10)	<0.001	1.90 (1.68–2.15)	<0.001	1.91 (1.69–2.16)	<0.001
*High frailty*	3.02 (2.70–3.38)	<0.001	2.76 (2.44–3.11)	<0.001	2.87 (2.54–3.25)	<0.001	2.90 (2.56–3.29)	<0.001
*Very high frailty*	4.63 (4.13–5.19)	<0.001	4.01 (3.52–4.57)	<0.001	4.22 (3.69–4.82)	<0.001	4.34 (3.76–5.01)	<0.001
*P*-*value or trend*	1.81 (1.75–1.89)	<0.001	1.68 (1.61–1.76)	<0.001	1.71 (1.63–1.79)	<0.001	1.74 (1.65–1.83)	<0.001

CI – confidence interval, ELSA – English Longitudinal Study of Ageing, FI – Frailty Index, HRS – Health and Retirement Study, ref – reference, SHARE – Survey of Health, Ageing and Retirement in Europe, sHR – subdistribution hazard ratio

*FI was analysed on a percentage scale (0–100). HRs and sHRs for FI represent the relative risk associated with a one-percentage-point increase in FI. Model 1 was the crude model. Model 2 was adjusted for age, sex, race, education, marital status, wealth, and BMI. Model 3 was further adjusted from model 2 for hypertension, diabetes, drinking status, and smoking status. Model 4 was further adjusted from model 3 for physical activity, cardiovascular disease, and cancer.

### Discrete-time logistic regression analysis

Discrete-time logistic regression models using follow-up waves as the time unit yielded results consistent with time-to-event analyses. In fully adjusted models, each one-percentage-point increase in FI was positively associated with new-onset osteoporosis, with aORs of 1.45 in the HRS (95% CI = 1.38–1.53, *P* < 0.001) and 1.40 in the SHARE (95% CI = 1.34–1.47, *P* < 0.001). Higher frailty trajectories were also associated with a higher risk of new-onset osteoporosis. These findings were consistent in both direction and magnitude with those from Cox and competing risk models (**Table 2**). Due to limited sample size, this analysis was not performed in the ELSA cohort.

**Table 2:** Discrete-time logistic regression analysis evaluating the associations between the FI, its trajectories, and the risk of new-onset osteoporosis*

	Model 1		Model 2		Model 3		Model 4	
	**OR (95% CI)**	***P*-value**	**OR (95% CI)**	***P*-value**	**OR (95% CI)**	***P*-value**	**OR (95% CI)**	***P*-value**
**HRS**								
Frailty	1.34 (1.29–1.40)	<0.001	1.37 (1.31–1.43)	<0.001	1.42 (1.36–1.49)	<0.001	1.45 (1.38–1.53)	<0.001
Frailty group								
*Low frailty*	ref		ref		ref		ref	
*Moderate frailty*	1.40 (1.21–1.63)	<0.001	1.55 (1.33–1.81)	<0.001	1.66 (1.42–1.94)	<0.001	1.67 (1.43–1.96)	<0.001
*High frailty*	1.95 (1.70–2.25)	<0.001	2.27 (1.95–2.65)	<0.001	2.54 (2.17–2.99)	<0.001	2.61 (2.22–3.08)	<0.001
*Very high frailty*	3.13 (2.72–3.61)	<0.001	3.64 (3.11–4.27)	<0.001	4.19 (3.55–4.96)	<0.001	4.40 (3.69–5.25)	<0.001
*P*-*value for trend*	1.62 (1.53–1.71)	<0.001	1.70 (1.60–1.80)	<0.001	1.79 (1.68–1.90)	<0.001	1.83 (1.71–1.95)	<0.001
**SHARE**								
Frailty	1.51 (1.46–1.56)	<0.001	1.40 (1.35–1.45)	<0.001	1.41 (1.36–1.47)	<0.001	1.40 (1.34–1.47)	<0.001
Frailty group								
*Low frailty*	ref		ref		ref		ref	
*Moderate frailty*	2.01 (1.77–2.30)	<0.001	1.97 (1.73–2.26)	<0.001	2.04 (1.78–2.34)	<0.001	2.05 (1.79–2.35)	<0.001
*High frailty*	3.39 (2.99–3.84)	<0.001	3.14 (2.74–3.59)	<0.001	3.31 (2.89–3.80)	<0.001	3.34 (2.90–3.84)	<0.001
*Very high frailty*	6.21 (5.46–7.06)	<0.001	5.51 (4.77–6.38)	<0.001	5.87 (5.05–6.83)	<0.001	5.95 (5.07–6.99)	<0.001
*P*-value for trend	2.05 (1.96–2.15)	<0.001	1.93 (1.82–2.03)	<0.001	1.96 (1.85–2.07)	<0.001	1.98 (1.87–2.11)	<0.001

CI – confidence interval, OR – odds ratio, ELSA – English Longitudinal Study of Ageing, FI – Frailty Index, HRS – Health and Retirement Study, ref – reference, SHARE – Survey of Health, Ageing and Retirement in Europe

*FI was analysed on a percentage scale (0–100). HRs and sHRs for FI represent the relative risk associated with a one-percentage-point increase in FI. Model 1 was the crude model. Model 2 was adjusted for age, sex, race, education, marital status, wealth, and BMI. Model 3 was further adjusted from model 2 for hypertension, diabetes, drinking status, and smoking status. Model 4 was further adjusted from model 3 for physical activity, cardiovascular disease, and cancer.

### Subgroup analyses

Subgroup analyses demonstrated consistent positive associations between FI and new-onset osteoporosis across strata defined by sex, age, and physical activity in the HRS and SHARE cohorts. In the ELSA cohort, stronger associations were observed among participants without cancer (*P* < 0.001) and those without diabetes (*P* < 0.001). Significant interactions were identified in the HRS cohort for age (*P*-value for interaction ≤ 0.001), sex (*P*-value for interaction ≤ 0.008), and physical activity (*P*-value for interaction = 0.008). No significant interactions were observed in the ELSA cohort. In the SHARE cohort, significant interactions were detected for age, sex, smoking status, physical activity, and cardiovascular disease, with all associated *P*-values for interaction <0.001 (**Figure 2**).

**Figure 2:**
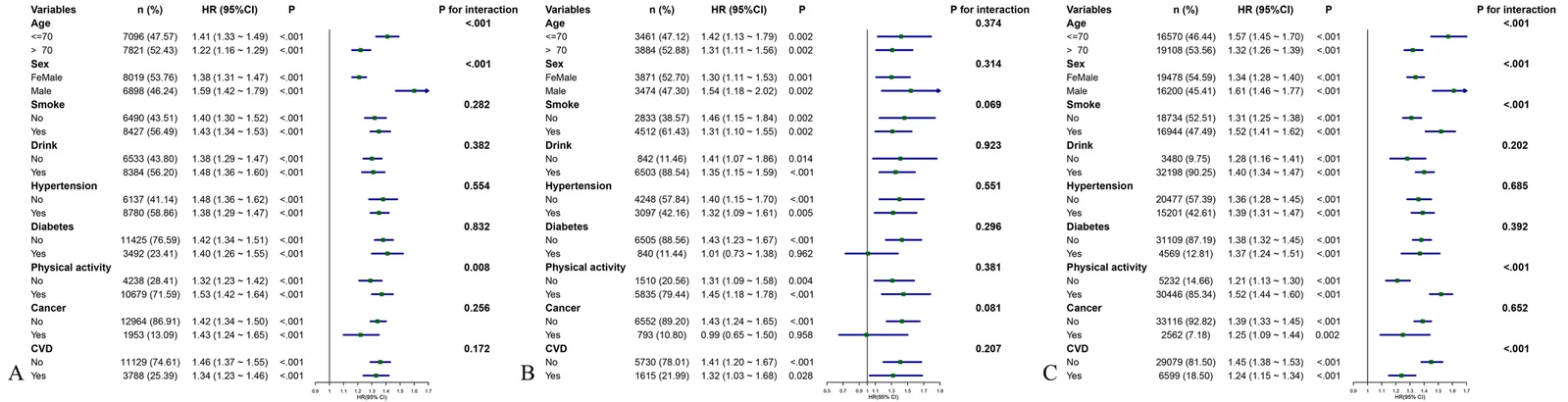
Forest plots showing the associations between the frailty index and the risk of new-onset osteoporosis across different subgroups in each cohort. **Panel A.** HRS cohort. **Panel B.** ELSA cohort. **Panel C.** SHARE cohort.

### Linear and nonlinear associations between FI and new-onset osteoporosis

Restricted cubic spline analyses revealed significant nonlinear associations between FI and new-onset osteoporosis in unadjusted models across all cohorts, with all *P*-values for nonlinearity <0.001). After adjustment, nonlinearity persisted in the HRS (*P*-value for nonlinearity <0.001) and SHARE cohorts (*P*-value for nonlinearity <0.001), while the association appeared linear in the ELSA cohort (**Figure 3**). In terms of the subsequent threshold analysis, we identified an inflection point was identified in the HRS cohort at a FI of 25 (equivalent to a conventional FI value of 0.25). Below this threshold, each one-percentage-point increase in FI (equivalent to a 0.01 increase on the conventional FI scale) was associated with a 4.8% higher risk (HR = 1.048; 95% CI = 1.040–1.057, *P* < 0.001). Above the threshold, the association remained significant, but was attenuated (HR = 1.014; 95% CI = 1.009–1.019, *P* < 0.001). Model fit was significantly improved using a two-piece linear model (*P* < 0.001). The inflection point in the SHARE cohort was 35.776 (equivalent to a conventional FI value of 0.35776). Below this value, each one-percentage-point increase in FI (equivalent to a 0.01 increase on the conventional FI scale) was associated with a 4.1% higher risk (HR = 1.041; 95% CI = 1.036–1.046, *P* < 0.001). Above the threshold, the association was no longer significant (HR = 1.003; 95% CI = 0.995–1.011, *P* = 0.487). These findings indicate a nonlinear association, with a steeper increase in risk at lower FI levels and a plateau at higher levels (Table S8 in the **Online Supplementary Document**).

**Figure 3:**
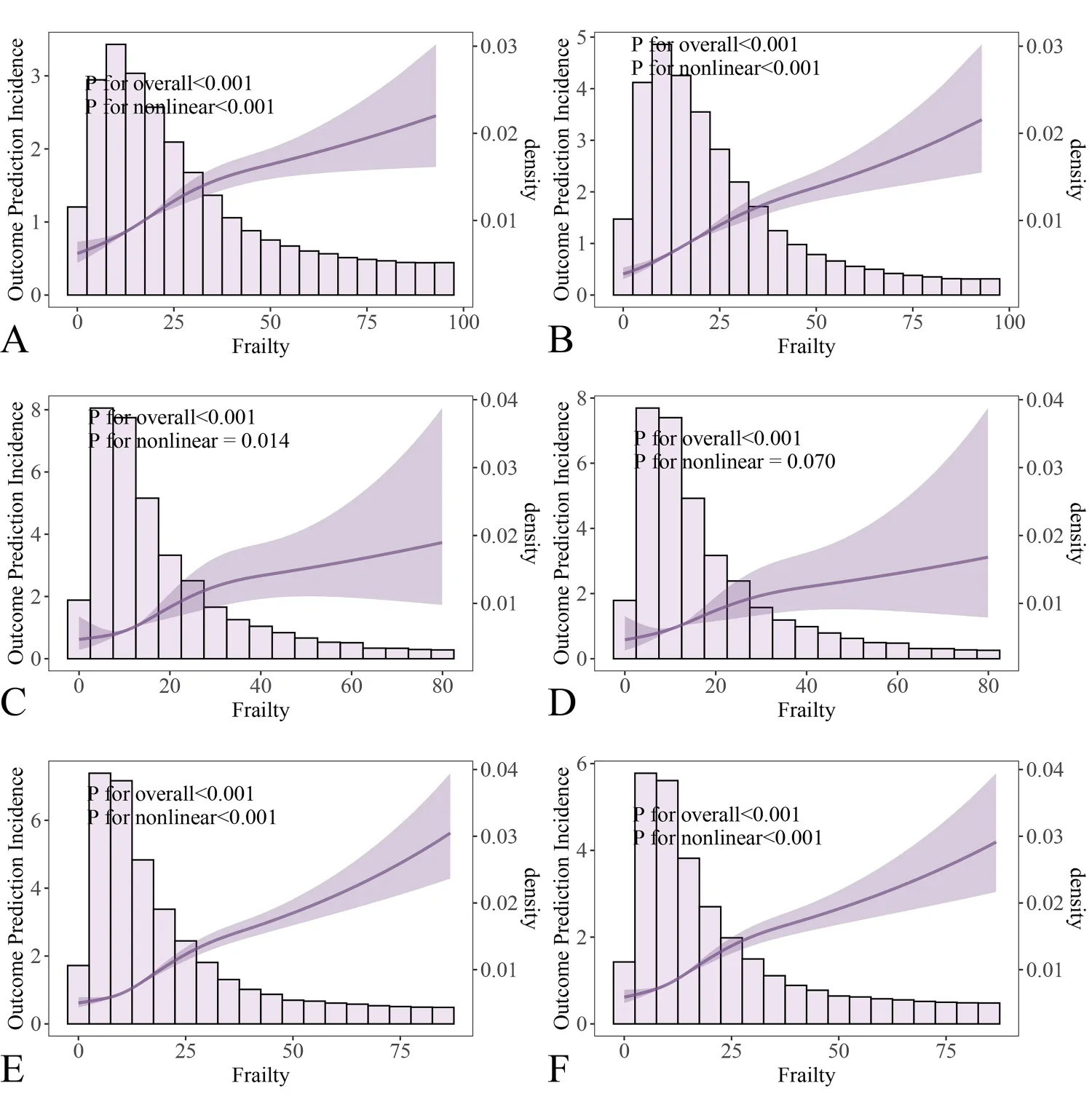
Restricted cubic spline analyses illustrating both linear and nonlinear associations between the frailty index and the risk of new-onset osteoporosis. **Panel A.** Unadjusted model in the HRS cohort. **Panel B.** Fully adjusted model in the HRS cohort. **Panel C.** Unadjusted model in the ELSA cohort. **Panel D.** Fully adjusted model in the ELSA cohort. **Panel E.** Unadjusted model in the SHARE cohort. **Panel F.** Fully adjusted model in the SHARE cohort.

### Mediation analysis

Mediation analysis (Figure S4 in the **Online Supplementary Document**) indicated that muscle strength, assessed by grip strength, partially mediated the association between FI and new-onset osteoporosis. The proportion of the association between FI and new-onset osteoporosis mediated through grip strength was 10.0% (95% CI = 7.1–14.0, *P* < 0.001) in the HRS cohort and 14.0% (95% CI = 8.9–18.0, *P* < 0.001) in the SHARE cohort. We did not perform the mediation analysis in the ELSA cohort due to the absence of grip strength data.

## DISCUSSION

We used data from three large international longitudinal cohorts – the HRS, the ELSA, and the SHARE – to systematically examine the associations between the FI, its dynamic trajectories, and the risk of new-onset osteoporosis. Several key findings emerged. First, baseline FI was independently and positively associated with new-onset osteoporosis across all cohorts. Second, trajectory analyses identified distinct frailty patterns, demonstrating clear risk stratification; individuals with higher baseline frailty or more rapid progression generally exhibited an increased risk of new-onset osteoporosis across cohorts. Third, nonlinear analyses revealed threshold effects in the HRS and SHARE cohorts, with risk increasing more sharply at lower FI levels and tending to plateau at higher levels, while the association appeared approximately linear in the ELSA cohort. Furthermore, multiple sensitivity analyses, including competing-risk and discrete-time models, generally supported the robustness of the observed associations, although an irregular pattern was noted in the ELSA competing-risk analysis and is discussed below. Finally, mediation analyses in the HRS and SHARE cohorts indicated that grip strength partially mediated the association between frailty and osteoporosis. Despite some cohort-specific differences in secondary analyses, these results provide converging evidence on how frailty is linked to osteoporosis from both static and dynamic perspectives, offering important insights into underlying mechanisms and potential early intervention strategies.

Previous studies have primarily relied on cross-sectional designs or single time point assessments of FI. These investigations consistently demonstrated associations between frailty and reduced bone mineral density, osteoporotic fractures, and other adverse outcomes [23–25]. Longitudinal evidence has further linked baseline frailty to incident fractures and accelerated functional decline following osteoporotic events. However, only a few studies have applied group-based trajectory modelling to capture within-individual heterogeneity or to explore nonlinear dose–response relationships [26,27]. Our multi-cohort analysis extends prior research by employing a prospective, trajectory-based framework and confirming consistent associations between FI and osteoporosis across populations drawn from North America and Europe. Although the overall direction of associations was highly consistent, some quantitative differences in effect estimates and trajectory distributions were observed across cohorts. Such heterogeneity is not unexpected given the distinct characteristics of the HRS, ELSA, and SHARE populations, which represent older adults in the USA, the ageing population of England, and participants from multiple European countries with diverse healthcare systems and social environments, respectively. Differences in osteoporosis screening practices, access to healthcare services, demographic composition, socioeconomic conditions, and baseline frailty burden may influence both the prevalence of frailty and the probability of osteoporosis diagnosis. Therefore, modest between-cohort variation should be interpreted within the context of these population-level differences rather than as evidence of inconsistent associations. In addition, we quantified the mediating role of muscle strength and identified a threshold effect, thereby enhancing mechanistic understanding and clinical relevance beyond previous single-cohort or phenotype-based studies [28–30].

A key mechanism underlying the observed association involves the disruption of the muscle–bone axis. Progressive sarcopenia in frail individuals reduces mechanical loading on bone, thereby decreasing osteoblast activity and increasing net bone loss [31,32]. Beyond mechanical loading, muscle and bone also interact through biochemical signalling pathways. Frailty-related muscle atrophy may impair this muscle–bone crosstalk, thereby contributing to osteoporosis development [33]. In our present study, grip strength – a robust proxy for overall muscle function – partially mediated the frailty–new-onset osteoporosis association. This finding aligns with evidence that preservation of muscle strength can attenuate bone loss, even in the context of ageing [34,35].

A second pathway involves chronic low-grade inflammation associated with frailty. Elevated circulating levels of proinflammatory cytokines, including interleukin-6 and tumour necrosis factor *α*, were found to be strongly correlated with higher FI scores; these cytokines promote osteoclastogenesis, while inhibiting osteoblast differentiation, thereby shifting bone remodelling toward resorption [36,37]. Concurrently, this proinflammatory state may, therefore, contribute simultaneously to worsening frailty and accelerated bone loss [38].

Endocrine and metabolic dysregulation represents a third interconnected pathway potentially linking frailty with osteoporosis. Age-related declines in oestrogen, testosterone, growth hormone, and insulin-like growth factor-1 – more pronounced in frail individuals – impair osteoblast function and enhance osteoclast activity. Simultaneously, vitamin D deficiency and disrupted calcium homeostasis further compromise bone mineralisation [39,40]. Collectively, endocrine and metabolic alterations associated with frailty may further accelerate skeletal deterioration and osteoporosis development.

The identified nonlinear and threshold effects provide important clinical insights. At mild-to-moderate levels of frailty, residual physiological reserves may permit compensatory responses, including increased physical activity or hormonal regulation. However, as frailty progresses, these compensatory mechanisms become overwhelmed, resulting in a steep increase in osteoporosis risk [41]. Beyond the threshold, a plateau effect may emerge, potentially reflecting survival bias, competing mortality, or depletion of modifiable biological pathways. These findings suggest that early to moderate stages of frailty represent a critical window for preventive intervention [12,13].

From a clinical and public health perspective, routine frailty screening – particularly when integrated into electronic health record systems – offers a cost-effective and scalable strategy for identifying individuals at high risk of osteoporosis. Compared with single-time point assessments, trajectory-based monitoring provides a more nuanced understanding of risk evolution over time [14,15]. Dynamic evaluation of FI may inform personalised interventions, including resistance exercise, protein supplementation, and anti-inflammatory lifestyle modifications, aimed at preserving muscle strength and disrupting the muscle–bone–inflammation axis [6,8]. In resource-limited settings, such approaches may substantially reduce fracture incidence and associated disability, aligning with current priorities for frailty-centred care in ageing populations [42].

This study has several strengths, including its large multinational sample, prospective design with repeated FI measurements, application of group-based trajectory modelling, and robust validation using multiple analytical approaches, including competing risk models. The incorporation of nonlinear and threshold analyses, along with formal mediation testing, further strengthens the findings. However, several limitations should be acknowledged. The use of self-reported osteoporosis diagnoses may have introduced misclassification bias. We also had to exclude many participants who lacked follow-up information or data required for frailty assessment. Comparison of baseline characteristics between included and excluded participants showed that excluded individuals were generally older, frailer, less physically active, and had a higher burden of chronic conditions. Therefore, potential selection bias resulting from attrition and missing data cannot be completely excluded. Because participants with poorer health status and higher frailty were more likely to be excluded, the observed association between frailty and incident osteoporosis may have been underestimated rather than overestimated. In addition, frailty trajectories were derived from the first three study waves, and follow-up for incident osteoporosis began only after completion of the trajectory assessment period. Although this design strengthened temporal ordering between frailty trajectories and subsequent osteoporosis risk, it required participants to remain osteoporosis-free and under observation during the trajectory ascertainment phase. Consequently, individuals who developed osteoporosis, died, or were lost to follow-up before trajectory classification were not included in the trajectory analyses, potentially introducing survivor bias and selective attrition. Because participants with poorer health status and more severe frailty are more likely to experience early events or dropout, the estimates of the associations between adverse frailty trajectories and incident osteoporosis may be conservative.

Residual confounding from unmeasured variables also cannot be completely excluded, since several established osteoporosis-related risk factors were not consistently available or harmonisable across the three cohorts, including medication use (particularly corticosteroids and bone-protective therapies such as bisphosphonates, denosumab, and teriparatide), hormone replacement therapy, menopausal status and time since menopause, dietary calcium intake, vitamin D intake, and bone-specific biomarkers. These factors may influence both frailty and osteoporosis risk and, therefore, represent potential sources of residual confounding. For example, individuals with greater frailty may be more likely to receive corticosteroid treatment, have poorer nutritional status, lower calcium and vitamin D intake, and experience age-related hormonal changes that adversely affect bone health. Consequently, the observed association between frailty and incident osteoporosis may have been partially overestimated. Although we applied a harmonised deficit accumulation framework across all cohorts, some differences in the availability and composition of health deficit variables were unavoidable. Therefore, residual variability in FI construction across cohorts cannot be completely excluded. In addition, the study population consisted primarily of community-dwelling older adults from the USA and Europe. Consequently, caution is warranted when extrapolating these findings to younger populations, institutionalised individuals, or populations from other geographic and healthcare settings. An additional point requiring cautious interpretation is the competing-risk analysis in the ELSA cohort. Although most analyses demonstrated a graded increase in osteoporosis risk across frailty trajectory groups, the Fine–Gray model showed a higher sHR for the moderate frailty group than for the high frailty group. We observed this pattern only in the ELSA, and it was not replicated in the primary Cox models or in the HRS and SHARE cohorts. Given the relatively small number of osteoporosis events in ELSA and the further subdivision of participants into multiple trajectory groups, random variation and limited statistical precision may have contributed to this apparent inconsistency. Therefore, this isolated finding should be interpreted cautiously and does not materially alter the overall evidence supporting a positive association between frailty trajectories and incident osteoporosis. The absence of grip strength data in the ELSA cohort limited the generalisability of mediation analyses.

Furthermore, causal inference from our analyses is constrained by the observational design of our study. The FI should be viewed as a composite indicator of overall health deficit burden, rather than a single causal exposure. Therefore, our findings should be interpreted as demonstrating a graded association between frailty burden and incident osteoporosis risk, rather than indicating that the FI itself acts as a singular causal agent. Finally, the mediation analyses were conducted within an observational framework and, therefore, depend on assumptions regarding the absence of unmeasured exposure–mediator, exposure–outcome, and mediator–outcome confounding. Although we adjusted for a broad range of potential confounders, causal interpretations of the mediated effects should be made cautiously. Future studies targeting potentially modifiable components of frailty and related biological pathways are needed to better clarify causal mechanisms and identify effective strategies for osteoporosis prevention.

## CONCLUSIONS

Based on large-scale multinational longitudinal data, we observed a robust and consistent association between the FI, its dynamic trajectories, and the risk of new-onset osteoporosis, with clear nonlinear trends, and with grip strength partially mediating this relationship. These findings underscore the importance of early identification and longitudinal monitoring of frailty in ageing populations and suggest that interventions targeting muscle strength may offer a promising strategy for osteoporosis prevention.

## Additional material


Online Supplementary Document

## Data Availability

**Data availability:** The data used in the present study were obtained from publicly accessible, deidentified datasets available through the official cohort platforms – the HRS (https://hrs.isr.umich.edu/), the ELSA (https://www.elsa-project.ac.uk/), and the SHARE (https://share-eric.eu/) – upon application and approval according to their respective data access policies.
