# Supplementary material for: Beyond static frailty: longitudinal trajectories, nonlinear associations, and muscle strength mediation in osteoporosis risk
Source: J Glob Health. 2026 Oct 19;16:04320. doi: 10.7189/jogh.16.04320 (PMC13679352; doi:10.7189/jogh.16.04320)
Supplement: Supplementary file 1 [file jogh-16-04320-s001.pdf]

Supplement to: Zhou J, Wang J, Huang Z, Cao L. Beyond static frailty: longitudinal trajectories, nonlinear associations, and muscle strength mediation in osteoporosis risk. J Glob Health. 2026;16:04320.

Table S1. Health domains and deficit categories included in frailty index construction across the HRS, ELSA, and SHARE cohorts.

Domain	Representative deficits	Coding
Chronic diseases	Hypertension, diabetes, heart disease, stroke, cancer, chronic lung disease, arthritis and other physician-diagnosed conditions	0 = absence, 1 = presence
Functional limitations	Difficulty walking, climbing stairs, lifting or carrying objects, pushing or pulling large objects	0 = no limitation, 1 = limitation
Activities of daily living (ADL)	Bathing, dressing, eating, transferring, toileting	0 = independent, 1 = dependent
Instrumental activities of daily living (IADL)	Shopping, meal preparation, managing money, telephone use, medication management	0 = independent, 1 = dependent
Self-rated health	Self-rated health status	0–1 scale
Cognitive function	Memory and cognitive performance indicators	0–1 scale
Psychological status	Depressive symptoms and emotional well-being	0–1 scale
Sensory impairment	Vision and hearing difficulties	0 = absent, 1 = present

Abbreviations: ADL, activities of daily living; IADL, instrumental activities of daily living.

The frailty index (FI) was constructed using the deficit accumulation approach proposed by Rockwood and Mitnitski and followed previously validated protocols applied in ageing cohort studies. Deficits encompassed multiple health domains, including chronic diseases, functional limitations, activities of daily living, instrumental activities of daily living, self-rated health, cognitive function, psychological well-being, and sensory impairments. Each deficit was coded on a scale from 0 to 1, and the FI score was calculated as the proportion of deficits present among all available deficits. For ordinal variables, proportional coding was applied according to the severity and ordering of response categories. Intermediate response levels were assigned values between 0 and 1, with higher values indicating greater deficit burden. Conceptually equivalent variables were harmonized across the HRS, ELSA, and SHARE cohorts whenever possible. When exact correspondence was unavailable, the closest validated equivalent measure was selected to maximize comparability across cohorts.

Table S2. Baseline characteristics of participants stratified by new-onset osteoporosis in the HRS cohort.

**Characteristic**	**N**	**Overall**<br>N = 14,917	**Normal**<br>N = 12,931	**New-onset Osteoporosis**<br>N = 1,986	**p-value**
Sex[b], n(%)	14,917				<0.001
Female		8,019 (53.76%)	6,390 (49.42%)	1,629 (82.02%)	
Male		6,898 (46.24%)	6,541 (50.58%)	357 (17.98%)	
Age(year)[a], M (Q1, Q3)	14,917	64.00 (57.00 - 74.00)	64.00 (57.00 - 74.00)	67.00 (59.00 - 76.00)	<0.001
Race[b], n(%)	14,917				<0.001
Non-Hispanic white		9,371 (62.82%)	8,007 (61.92%)	1,364 (68.68%)	
Non-Hispanic Blacks		3,054 (20.47%)	2,759 (21.34%)	295 (14.85%)	
Hispanic		2,008 (13.46%)	1,739 (13.45%)	269 (13.54%)	
Other Race		484 (3.24%)	426 (3.29%)	58 (2.92%)	
Education[b], n(%)	14,917				0.522
High school or less		3,058 (20.50%)	2,665 (20.61%)	393 (19.79%)	
Some college		8,173 (54.79%)	7,062 (54.61%)	1,111 (55.94%)	
College graduate or above		3,686 (24.71%)	3,204 (24.78%)	482 (24.27%)	
Wealth[a], M (Q1, Q3)	14,917	135,000.00 (18,500.00 - 433,000.00)	133,400.00 (18,600.00 - 427,000.00)	150,000.00 (18,000.00 - 475,000.00)	0.046
BMI[a], M (Q1, Q3)	14,917	28.00 (24.70 - 32.00)	28.10 (24.90 - 32.00)	27.10 (23.80 - 31.70)	<0.001
Hypertension[b], n(%)	14,917				0.128
No		6,137 (41.14%)	5,351 (41.38%)	786 (39.58%)	
Yes		8,780 (58.86%)	7,580 (58.62%)	1,200 (60.42%)	
Diabetes[b], n(%)	14,917				0.003
No		11,425 (76.59%)	9,851 (76.18%)	1,574 (79.25%)	

Characteristic	N	Overall<br>N = 14,917	Normal<br>N = 12,931	New-onset Osteoporosis<br>N = 1,986	p-value
Yes		3,492 (23.41%)	3,080 (23.82%)	412 (20.75%)	
Smoking status[b], n(%)	14,917				<0.001
No		6,490 (43.51%)	5,513 (42.63%)	977 (49.19%)	
Yes		8,427 (56.49%)	7,418 (57.37%)	1,009 (50.81%)	
Drinking status[b], n(%)	14,917				<0.001
No		6,533 (43.80%)	5,531 (42.77%)	1,002 (50.45%)	
Yes		8,384 (56.20%)	7,400 (57.23%)	984 (49.55%)	
Cancer [b], n(%)	14,917				0.038
No		12,964 (86.91%)	11,267 (87.13%)	1,697 (85.45%)	
Yes		1,953 (13.09%)	1,664 (12.87%)	289 (14.55%)	
Physical activity [b], n(%)	14,917				<0.001
No		4,238 (28.41%)	3,587 (27.74%)	651 (32.78%)	
Yes		10,679 (71.59%)	9,344 (72.26%)	1,335 (67.22%)	
Cardiovascular disease	14,917				<0.001
No		11,129 (74.61%)	9,713 (75.11%)	1,416 (71.30%)	
Yes		3,788 (25.39%)	3,218 (24.89%)	570 (28.70%)	
Grip strength [a], M (Q1, Q3)	14,917	30.50 (24.00 - 40.00)	31.50 (24.50 - 41.00)	25.00 (20.50 - 31.00)	<0.001
Frailty [a], M (Q1, Q3)	14,917	16.67 (9.17 - 27.50)	16.15 (9.17 - 26.67)	21.80 (12.50 - 33.99)	<0.001
Frailty group [b], n(%)	14,917				<0.001
Low frailty		3,693 (24.76%)	3,401 (26.30%)	292 (14.70%)	
Moderate frailty		4,280 (28.69%)	3,815 (29.50%)	465 (23.41%)	
High frailty		4,055 (27.18%)	3,462 (26.77%)	593 (29.86%)	
Very high frailty		2,889 (19.37%)	2,253 (17.42%)	636 (32.02%)	

Characteristic	N	Overall<br>N = 14,917	Normal<br>N = 12,931	New-onset Osteoporosis<br>N = 1,986	p-value

a: Kruskal-Wallis rank sum test, b: Chi-square test

Table S3. Baseline characteristics of participants stratified by new-onset osteoporosis in the ELSA cohort.

Characteristic	N	Overall<br>N = 7,345	Normal<br>N = 7,101	New-onset Osteoporosis<br>N = 244	p-value
Sex[b], n(%)	7,345				<0.001
Female		3,871 (52.70%)	3,692 (51.99%)	179 (73.36%)	
Male		3,474 (47.30%)	3,409 (48.01%)	65 (26.64%)	
Age(year)[a], M (Q1, Q3)	7,345	66.00 (60.00 - 73.00)	66.00 (60.00 - 73.00)	69.50 (62.00 - 77.00)	<0.001
Race[b], n(%)	7,345				0.413
No white		261 (3.55%)	250 (3.52%)	11 (4.51%)	
White		7,084 (96.45%)	6,851 (96.48%)	233 (95.49%)	
Education[b], n(%)	7,345				<0.001
Before high school		2,226 (30.31%)	2,122 (29.88%)	104 (42.62%)	
High school		1,698 (23.12%)	1,642 (23.12%)	56 (22.95%)	
Junior college		1,878 (25.57%)	1,823 (25.67%)	55 (22.54%)	
College graduate or above		1,543 (21.01%)	1,514 (21.32%)	29 (11.89%)	
Wealth[a], M (Q1, Q3)	7,345	272,000.00 (136,400.00 - 487,153.00)	273,100.00 (138,100.00 - 490,000.00)	218,100.00 (81,500.00 - 425,834.50)	0.002
Hypertension[b], n(%)	7,345				0.681
No		4,248 (57.84%)	4,110 (57.88%)	138 (56.56%)	
Yes		3,097 (42.16%)	2,991 (42.12%)	106 (43.44%)	
Diabetes[b], n(%)	7,345				0.063

Characteristic	N	Overall<br>N = 7,345	Normal<br>N = 7,101	New-onset Osteoporosis<br>N = 244	p-value
No		6,505 (88.56%)	6,298 (88.69%)	207 (84.84%)	
Yes		840 (11.44%)	803 (11.31%)	37 (15.16%)	
Smoking status[b], n(%)	7,345				0.223
No		2,833 (38.57%)	2,748 (38.70%)	85 (34.84%)	
Yes		4,512 (61.43%)	4,353 (61.30%)	159 (65.16%)	
Drinking status[b], n(%)	7,345				<0.001
No		842 (11.46%)	794 (11.18%)	48 (19.67%)	
Yes		6,503 (88.54%)	6,307 (88.82%)	196 (80.33%)	
Cancer [b], n(%)	7,345				0.235
No		6,552 (89.20%)	6,340 (89.28%)	212 (86.89%)	
Yes		793 (10.80%)	761 (10.72%)	32 (13.11%)	
Physical activity [b], n(%)	7,345				<0.001
No		1,510 (20.56%)	1,424 (20.05%)	86 (35.25%)	
Yes		5,835 (79.44%)	5,677 (79.95%)	158 (64.75%)	
Cardiovascular disease [b], n(%)	7,345				0.104
No		5,730 (78.01%)	5,550 (78.16%)	180 (73.77%)	
Yes		1,615 (21.99%)	1,551 (21.84%)	64 (26.23%)	
Frailty [a], M (Q1, Q3)	7,345	11.84 (6.75 - 20.78)	11.64 (6.67 - 20.32)	18.38 (10.03 - 30.83)	<0.001
Frailty group [b], n(%)	7,345				<0.001
Low frailty		2,576 (35.07%)	2,538 (35.74%)	38 (15.57%)	
Moderate frailty		2,005 (27.30%)	1,954 (27.52%)	51 (20.90%)	
High frailty		1,523 (20.74%)	1,459 (20.55%)	64 (26.23%)	
Very high frailty		1,241 (16.90%)	1,150 (16.19%)	91 (37.30%)	

**Characteristic**	**N**	**Overall**<br>N = 7,345	**Normal**<br>N = 7,101	**New-onset Osteoporosis**<br>N = 244	**p-value**

a: Kruskal-Wallis rank sum test, b: Chi-square test

Table S4. Baseline characteristics of participants stratified by new-onset osteoporosis in the SHARE cohort.

**Characteristic**	**N**	**Overall** N = 35,678	**Normal** N = 32,985	**New-onset Osteoporosis** N = 2,693	**p-value**
Sex[b], n(%)	35,678				<0.001
Female		19,478 (54.59%)	17,188 (52.11%)	2,290 (85.04%)	
Male		16,200 (45.41%)	15,797 (47.89%)	403 (14.96%)	
Age(year)[a], M (Q1, Q3)	35,678	64.00 (58.00 - 72.00)	64.00 (57.00 - 72.00)	68.00 (61.00 - 75.00)	<0.001
Citizens of this country [b], n(%)	35,678				0.607
No		1,547 (4.34%)	1,425 (4.32%)	122 (4.53%)	
Yes		34,131 (95.66%)	31,560 (95.68%)	2,571 (95.47%)	
Education[b], n(%)	35,678				<0.001
Before high school		14,273 (40.01%)	12,984 (39.36%)	1,289 (47.86%)	
High school and vocational training		13,786 (38.64%)	12,871 (39.02%)	915 (33.98%)	
Higher Education		7,619 (21.35%)	7,130 (21.62%)	489 (18.16%)	
Wealth[a], M (Q1, Q3)	35,678	160,648.24 (45,416.63 - 350,300.82)	162,621.77 (46,304.99 - 353,590.96)	144,566.83 (35,151.41 - 306,000.00)	<0.001
BMI[a], M (Q1, Q3)	35,678	26.37 (23.88 - 29.39)	26.40 (23.92 - 29.39)	25.95 (23.14 - 29.38)	<0.001
Hypertension[b], n(%)	35,678				<0.001
No		20,477 (57.39%)	19,096 (57.89%)	1,381 (51.28%)	
Yes		15,201 (42.61%)	13,889 (42.11%)	1,312 (48.72%)	
Diabetes[b], n(%)	35,678				0.004
No		31,109 (87.19%)	28,809 (87.34%)	2,300 (85.41%)	
Yes		4,569 (12.81%)	4,176 (12.66%)	393 (14.59%)	

**Characteristic**	**N**	**Overall**<br>N = 35,678	**Normal**<br>N = 32,985	**New-onset Osteoporosis**<br>N = 2,693	**p-value**
Smoking status[b], n(%)	35,678				<0.001
No		18,734 (52.51%)	17,006 (51.56%)	1,728 (64.17%)	
Yes		16,944 (47.49%)	15,979 (48.44%)	965 (35.83%)	
Drinking status[b], n(%)	35,678				<0.001
No		3,480 (9.75%)	3,109 (9.43%)	371 (13.78%)	
Yes		32,198 (90.25%)	29,876 (90.57%)	2,322 (86.22%)	
Cancer[b], n(%)	35,678				<0.001
No		33,116 (92.82%)	30,671 (92.98%)	2,445 (90.79%)	
Yes		2,562 (7.18%)	2,314 (7.02%)	248 (9.21%)	
Physical activity[b], n(%)	35,678				<0.001
No		5,232 (14.66%)	4,669 (14.15%)	563 (20.91%)	
Yes		30,446 (85.34%)	28,316 (85.85%)	2,130 (79.09%)	
Cardiovascular disease[b], n(%)	35,678				<0.001
No		29,079 (81.50%)	27,027 (81.94%)	2,052 (76.20%)	
Yes		6,599 (18.50%)	5,958 (18.06%)	641 (23.80%)	
Grip strength[a], M (Q1, Q3)	35,678	60.00 (47.00 - 81.00)	62.00 (48.00 - 82.00)	48.00 (39.00 - 59.00)	<0.001
Frailty[a], M (Q1, Q3)	35,678	11.75 (6.75 - 20.29)	11.67 (6.67 - 19.71)	17.50 (10.00 - 28.33)	<0.001
Frailty group[b], n(%)	35,678				<0.001
Low frailty		13,129 (36.80%)	12,665 (38.40%)	464 (17.23%)	
Moderate frailty		9,457 (26.51%)	8,827 (26.76%)	630 (23.39%)	
High frailty		8,471 (23.74%)	7,590 (23.01%)	881 (32.71%)	
Very high frailty		4,621 (12.95%)	3,903 (11.83%)	718 (26.66%)	

a: Kruskal-Wallis rank sum test, b: Chi-square test

Table S5. Comparison of baseline characteristics between included and excluded participants in the HRS cohort

Variable	Excluded	Included	p	SMD
HRS				
n	5637	14917		
Age (mean (SD))	68.38 (14.11)	66.27 (10.43)	<0.001	0.171
Wealth (mean (SD))	347032.65 (1015678.33)	409040.51 (992700.05)	<0.001	0.062
BMI (mean (SD))	27.50 (6.42)	28.88 (6.12)	<0.001	0.221
Grip strength (mean (SD))	27.51 (10.37)	32.45 (11.00)	<0.001	0.463
Frailty (mean (SD))	28.40 (19.57)	20.18 (14.54)	<0.001	0.477
Sex = Male (%)	1642 (29.1)	6898 (46.2)	<0.001	0.359
Race (%)			<0.001	0.141
Non-Hispanic white	3769 (67.1)	9351 (62.9)		
Non-Hispanic Blacks	847 (15.1)	3038 (20.4)		
Hispanic	791 (14.1)	1983 (13.3)		
Other Race	209 (3.7)	484 (3.3)		
Education (%)			<0.001	0.133
High school or less	1334 (23.8)	3045 (20.5)		
Some college	3186 (56.7)	8132 (54.8)		
College graduate or above	1096 (19.5)	3665 (24.7)		
Smoking status = Yes (%)	3082 (55.0)	8385 (56.5)	0.054	0.030
Drinking status = Yes (%)	2633 (46.7)	8382 (56.2)	<0.001	0.191
Hypertension = Yes (%)	3423 (60.7)	8780 (58.9)	0.016	0.038
Diabetes = Yes (%)	1311 (23.3)	3492 (23.4)	0.832	0.004
Cancer = Yes (%)	1028 (18.2)	1953 (13.1)	<0.001	0.142

Variable	Excluded	Included	p	SMD
Cardiovascular disease = Yes (%)	1996 (35.4)	3788 (25.4)	<0.001	0.219
Physical activity = Yes (%)	3282 (58.4)	10659 (71.6)	<0.001	0.280
ELSA				
n	2321	7345		
Age (mean (SD))	68.48 (12.65)	66.93 (9.18)	<0.001	0.140
Wealth (mean (SD))	349735.80 (665325.24)	423905.50 (795609.84)	<0.001	0.101
Frailty (mean (SD))	22.15 (17.92)	15.67 (12.71)	<0.001	0.417
Sex = Male (%)	826 (35.6)	3474 (47.3)	<0.001	0.239
Race = White (%)	2194 (94.8)	7084 (96.4)	<0.001	0.082
Education (%)			<0.001	0.247
Before high school	852 (41.2)	2002 (30.0)		
High school	446 (21.6)	1541 (23.1)		
Junior college	444 (21.5)	1708 (25.6)		
College graduate or above	326 (15.8)	1423 (21.3)		
Smoking status = Yes (%)	1453 (64.8)	4446 (61.4)	0.004	0.070
Drinking status = Yes (%)	1336 (81.2)	5636 (88.3)	<0.001	0.198
Hypertension = Yes (%)	1020 (43.9)	3097 (42.2)	0.136	0.036
Diabetes = Yes (%)	308 (13.3)	840 (11.4)	0.019	0.056
Cancer = Yes (%)	345 (14.9)	793 (10.8)	<0.001	0.122
Cardiovascular disease = Yes (%)	709 (30.5)	1615 (22.0)	<0.001	0.195
Physical activity = Yes (%)	1439 (62.0)	5835 (79.4)	<0.001	0.391

Variable	Excluded	Included	p	SMD
SHARE				
n	22304	35678		
Age (mean (SD))	65.31 (11.45)	65.54 (9.65)	0.009	0.022
Wealth (mean (SD))	217949.58 (705620.35)	294106.88 (564259.60)	<0.001	0.119
BMI (mean (SD))	26.73 (4.81)	26.99 (4.70)	<0.001	0.055
Grip strength (mean (SD))	60.32 (23.54)	64.64 (23.42)	<0.001	0.184
Frailty (mean (SD))	19.23 (15.41)	15.42 (12.14)	<0.001	0.274
Sex = Male (%)	8878 (39.8)	16200 (45.4)	<0.001	0.113
Citizens of this country= Yes (%)	21192 (96.3)	33909 (95.7)	<0.001	0.032
Education (%)			<0.001	0.122
Before high school	10010 (44.9)	14273 (40.0)		
High school and vocational training	8471 (38.0)	13786 (38.6)		
Higher Education	3823 (17.1)	7619 (21.4)		
Smoking status = Yes (%)	9902 (45.7)	16704 (47.4)	<0.001	0.034
Drinking status = Yes (%)	16354 (85.0)	28805 (90.1)	<0.001	0.157
Hypertension = Yes (%)	9444 (42.9)	15201 (42.6)	0.485	0.006
Diabetes = Yes (%)	3052 (13.9)	4569 (12.8)	<0.001	0.031
Cancer = Yes (%)	1626 (7.4)	2562 (7.2)	0.352	0.008
Cardiovascular disease = Yes (%)	4676 (21.0)	6599 (18.5)	<0.001	0.062
Physical activity = Yes (%)	16514 (74.0)	30446 (85.3)	<0.001	0.284

Values are presented as mean ± SD for continuous variables and n (%) for categorical variables. Standardized mean differences (SMDs) were calculated to assess imbalance between included and excluded participants. SMD values <0.10 were considered

indicative of negligible imbalance.

Table S6. Associations between the frailty index, its trajectory patterns, and the risk of new-onset osteoporosis.

Exposure Variable	Model 1		Model 2		Model 3		Model 4	
	HR (95% CI)	P-value	HR (95% CI)	P-value	HR (95% CI)	P-value	HR (95% CI)	P-value
HRS								
Frailty	1.33 (1.28 ~ 1.38)	<.001***	1.35 (1.30 ~ 1.41)	<.001***	1.40 (1.34 ~ 1.46)	<.001***	1.43 (1.36 ~ 1.50)	<.001***
Frailty Group								
Low frailty	1.00 (Reference)	-	1.00 (Reference)	-	1.00 (Reference)	-	1.00 (Reference)	-
Moderate frailty	1.40 (1.21 ~ 1.62)	<.001***	1.54 (1.32 ~ 1.79)	<.001 ***	1.64 (1.40 ~ 1.91)	<.001***	1.65 (1.42 ~ 1.92)	<.001***
High frailty	1.94 (1.68 ~ 2.23)	<.001***	2.22 (1.91 ~ 2.58)	<.001 ***	2.48 (2.12 ~ 2.90)	<.001***	2.54 (2.16 ~ 2.98)	<.001***
Very high frailty	3.06 (2.67 ~ 3.52)	<.001***	3.48 (2.99 ~ 4.06)	<.001 ***	3.99 (3.39 ~ 4.69)	<.001***	4.18 (3.52 ~ 4.95)	<.001***
P for trend	1.60 (1.52 ~ 1.69)	<.001***	1.66 (1.57 ~ 1.76)	<.001 ***	1.75 (1.64 ~ 1.86)	<.001***	1.78 (1.67 ~ 1.90)	<.001***
ELSA								
Frailty	1.46 (1.33 ~ 1.61)	<.001***	1.36 (1.23 ~ 1.51)	<.001 ***	1.39 (1.24 ~ 1.55)	<.001***	1.36 (1.19 ~ 1.57)	<.001***
Frailty Group								
Low frailty	1.00 (Reference)	-	1.00 (Reference)	-	1.00 (Reference)	-	1.00 (Reference)	-
Moderate frailty	5.13 (3.52 ~ 7.50)	<.001***	4.19 (2.80 ~ 6.27)	<.001 ***	4.78 (3.11 ~ 7.33)	<.001***	4.89 (3.08 ~ 7.77)	<.001***
High frailty	1.73 (1.14 ~ 2.64)	0.010*	1.62 (1.06 ~ 2.47)	0.027 *	1.77 (1.15 ~ 2.71)	0.009**	1.80 (1.17 ~ 2.77)	0.007**
Very high frailty	2.88 (1.93 ~ 4.31)	<.001***	2.39 (1.58 ~ 3.61)	<.001 ***	2.69 (1.75 ~ 4.12)	<.001***	2.77 (1.79 ~ 4.28)	<.001***

P for trend	1.93 (1.68 ~ 2.21)	<.001***	1.78 (1.53 ~ 2.06)	<.001***	1.85 (1.58 ~ 2.16)	<.001***	1.86 (1.56 ~ 2.22)	<.001***
SHARE								
Frailty	1.50 (1.45 ~ 1.54)	<.001***	1.38 (1.34 ~ 1.43)	<.001***	1.40 (1.35 ~ 1.45)	<.001***	1.39 (1.33 ~ 1.45)	<.001***
Frailty Group								
Low frailty	1.00 (Reference)	-	1.00 (Reference)	-	1.00 (Reference)	-	1.00 (Reference)	-
Moderate frailty	1.96 (1.74 ~ 2.21)	<.001***	1.90 (1.68 ~ 2.15)	<.001***	1.96 (1.73 ~ 2.22)	<.001***	1.96 (1.73 ~ 2.22)	<.001***
High frailty	3.32 (2.97 ~ 3.72)	<.001***	3.03 (2.68 ~ 3.42)	<.001***	3.18 (2.81 ~ 3.60)	<.001***	3.19 (2.81 ~ 3.62)	<.001***
Very high frailty	5.89 (5.24 ~ 6.63)	<.001***	5.09 (4.47 ~ 5.81)	<.001***	5.41 (4.72 ~ 6.20)	<.001***	5.42 (4.68 ~ 6.26)	<.001***
P for trend	2.02 (1.93 ~ 2.10)	<.001***	1.87 (1.78 ~ 1.96)	<.001***	1.90 (1.81 ~ 2.00)	<.001***	1.91 (1.81 ~ 2.02)	<.001***

Model 1: Crude

Model 2: Adjust: Age, Sex, Race, Education, marital status, Wealth, BMI

Model 3: Adjust: Age, Sex, Race, Education, marital status, Wealth, BMI, Hypertension, Diabetes, Drinking status, Smoking status

Model 4: Adjust: Age, Sex, Race, Education, marital status, Wealth, BMI, Hypertension, Diabetes, Drinking status, Smoking status, Physical activity, Cardiovascular disease, Cancer

Significance: * $p < 0.05$, ** $p < 0.01$, *** $p < 0.001$

The frailty index (FI) was analyzed on a percentage scale (0–100). Hazard ratios for FI represent the relative risk associated with a one-percentage-point increase in FI.

Table S7. Evaluation indices of different group-based trajectory models (GBTM).

Model	AIC	BIC	Log-Likelihood
HRS			
1-class	439,649.5	439,695.1	-219,818.7
2-class	433,350.8	433,426.9	-216,665.4
3-class	431,421.4	431,527.9	-215,696.7
4-class	430,567.6	430,704.5	-215,265.8
Elsa			
1-class	158,603.8	158,645.2	-79,295.88
2-class	153,617.7	153,686.7	-76,798.86
3-class	152,607.2	152,703.8	-76,289.60
4-class	152,196.5	152,320.7	-76,080.23
Share			
1-class	1,044,174	1,044,225	-522,081.2
2-class	1,021,379	1,021,464	-510,679.7
3-class	1,016,043	1,016,162	-508,007.4
4-class	1,013,831	1,013,983	-506,897.3

Table S8. Threshold effect analysis assessing the nonlinear relationship between the frailty index and the risk of new-onset osteoporosis in the HRS and SHARE cohorts.

**Parameter**	**HR (95% CI), P-value**
HRS	
Model 1 Fitting model by standard linear regression	1.025(1.021-1.028), <0.001
Model 2 Fitting model by two-piecewise linear regression	
Inflection point	25
<25	1.048(1.04-1.057), <0.001
>25	1.014(1.009-1.019), <0.001
P for likelihood ratio test	<0.001
SHARE	
Model 1 Fitting model by standard linear regression	1.027(1.024-1.031), <0.001
Model 2 Fitting model by two-piecewise linear regression	
Inflection point	35.776
<35.776	1.041(1.036-1.046), <0.001
>35.776	1.003(0.995-1.011), 0.487
P for likelihood ratio test	<0.001

Model 1: Standard linear regression with Frailty as a continuous predictor.

Model 2: Two-piecewise linear regression with Frailty split at the inflection point.

Significance: $P < 0.05$ indicates statistical significance.

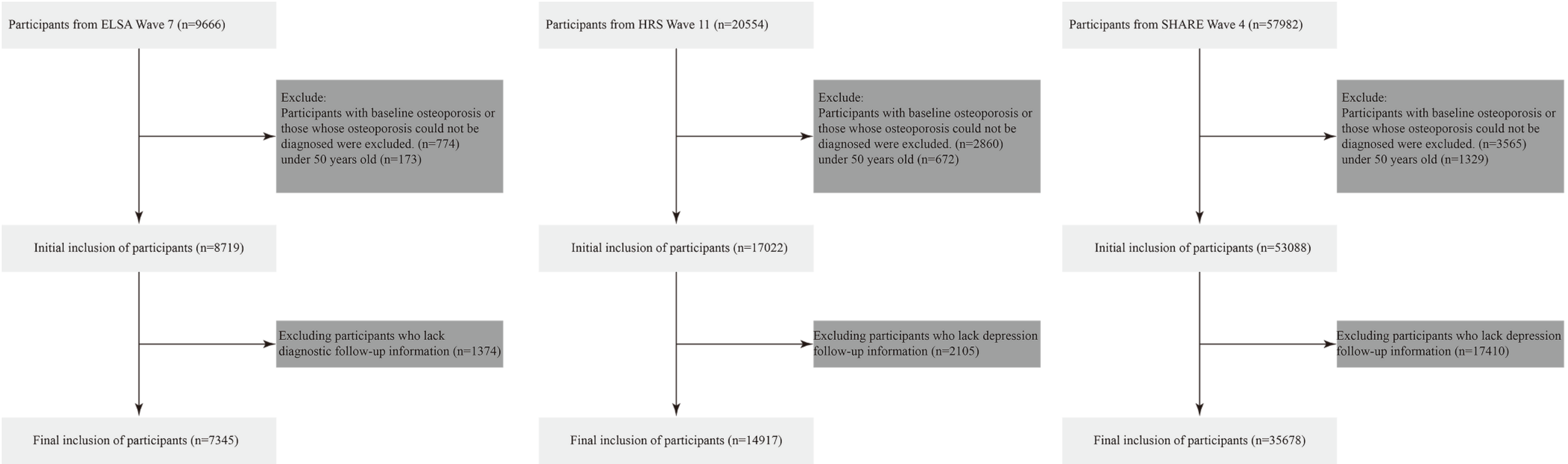

Figure S1. Flowchart of participant selection.

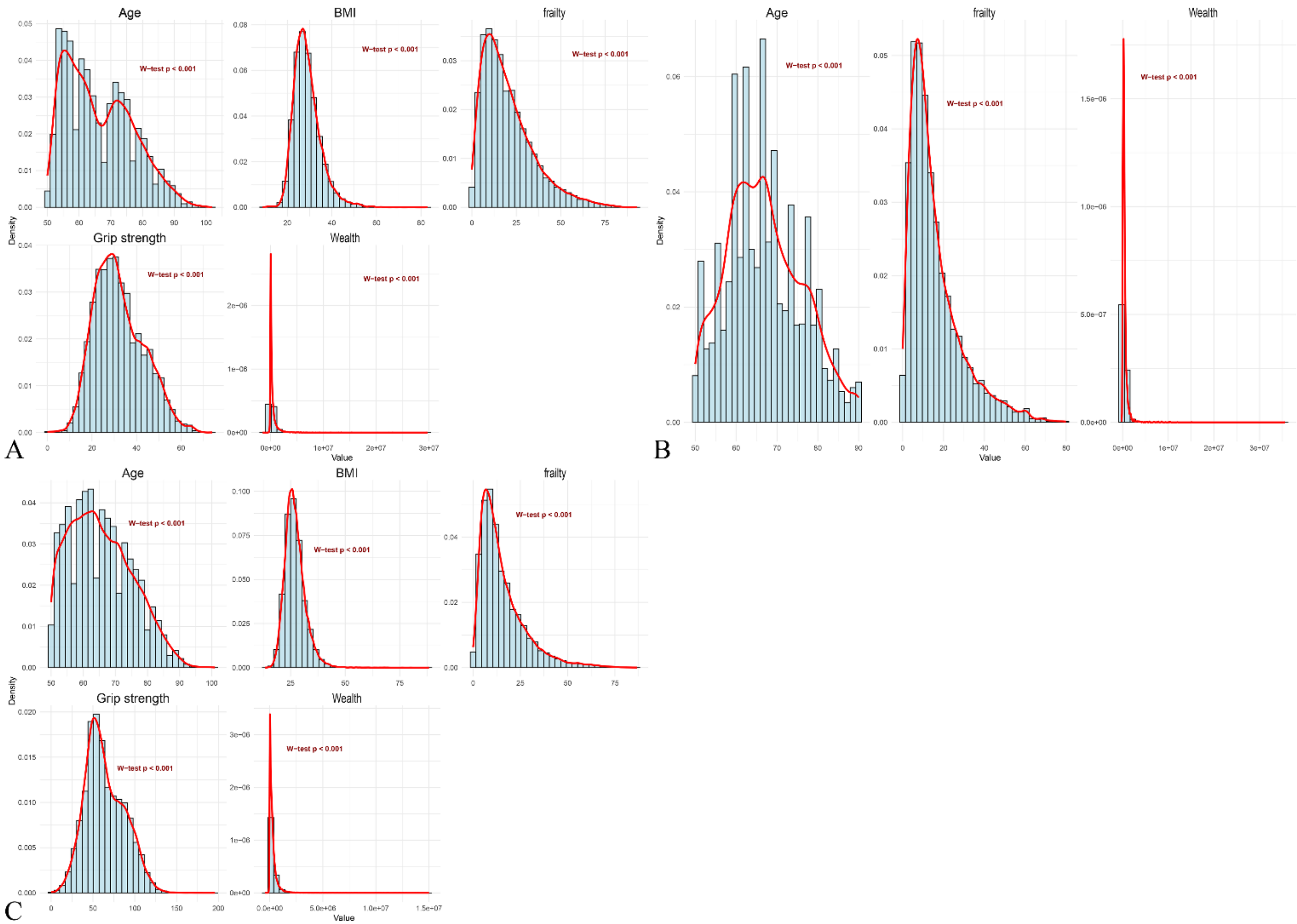

Figure S2. Distribution of continuous variables in the HRS, ELSA, and SHARE cohorts assessed using histograms with kernel density curves and Shapiro–Wilk tests.

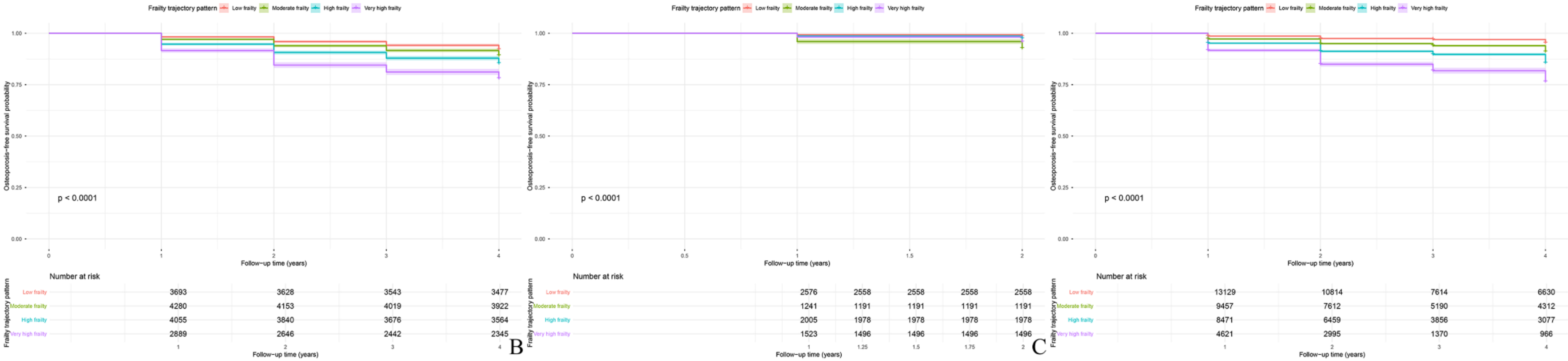

Figure S3. Kaplan–Meier curves illustrating differences in survival across frailty index trajectory groups: (A) HRS cohort; (B) ELSA cohort; (C) SHARE cohort.

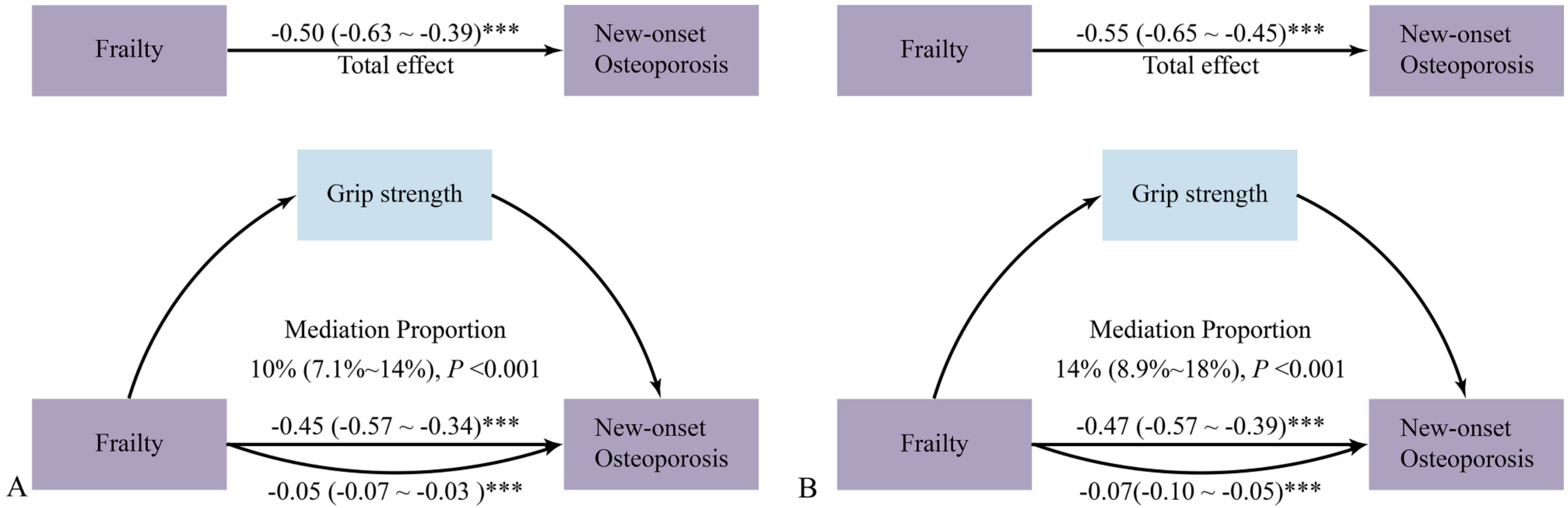

Figure S4. Mediation analysis examining the mediating role of muscle strength in the association between the frailty index and the risk of new-onset osteoporosis: (A) HRS cohort; (B) SHARE cohort.
